# The impact of medical financial assistance on healthcare expenses and the medical financial burden: Evidence from rural China

**DOI:** 10.3389/fpubh.2022.1021435

**Published:** 2023-01-19

**Authors:** Yucheng Chen, Gongjing Gao, Fei Yuan, Yuxiao Zhao

**Affiliations:** ^1^School of Political Science and Law, University of Jinan, Jinan, China; ^2^School of Medical Management, Shandong First Medical University, Jinan, Shandong, China

**Keywords:** medical financial assistance, medical financial burden, propensity score matching, China, health care expenses

## Abstract

**Background:**

The medical financial burden has become a key limitation to accessing healthcare in rural areas of China as healthcare expenses continue to rise. To ensure that low-income people have access to basic healthcare services, China has implemented medical financial assistance (MFA) policy, which provides social health insurance and medical cash assistance for low-income people.

**Methods:**

Using data from the 2014 China Family Panel Studies (CFPS), the propensity score matching (PSM) method was applied to estimate the impact of MFA on healthcare expenses and the medical financial burden.

**Results:**

Empirical results showed that the total annual healthcare expenditure of MFA beneficiaries is significantly higher than that of non-beneficiaries after matching. Although low-income individuals are now covered by MFA, neither the out-of-pocket expenditure to per capita household non-food expenditure ratio nor the likelihood of catastrophic healthcare expenditure (CHE) decrease significantly.

**Conclusion:**

Medical financial assistance (MFA) has reduced the inequality in healthcare utilization to a certain extent by improving access to healthcare for low-income people. However, people with low income still face a heavy medical financial burden even when they are covered by MFA. Policymakers should pay attention to raising the standards of MFA in rural areas and providing higher subsidies for the reasonable healthcare expenditures of low-income people.

## Highlights

- Financial barriers have become a key limitation to accessing healthcare for the poor in rural China in that healthcare expenses continue to rise.
- We evaluated the impact of medical financial assistance (MFA) on healthcare utilization and medical financial burden by using a nationally representative data.
- Medical financial assistance (MFA) released the healthcare needs of the poor and increased their healthcare expenditure.
- Medical financial assistance (MFA) did not significantly reduce the medical financial burden of the poor, this result is stable after considering the lag of policy implementation effect.

## Introduction

China is facing a surge in healthcare expenses. According to the China Health Statistics Yearbook, the total healthcare expenditure has increased from RMB ¥459 billion ($69 billion) to RMB ¥722 billion ($109 billion) in the past two decades. In 2020, personal healthcare expenditure also reached RMB ¥302 billion ($45 billion). This rapid increase in healthcare expenses will impose a huge financial burden on people (1, 2). It is well known that income is a critical factor associated with healthcare utilization (2, 3). In 2020, the disposable income of urban residents was approximately three times higher than that of rural residents. Therefore, rural residents face greater financial barriers to seeking healthcare than urban residents, which is more obvious in low-income population. People with low income usually have a greater need for healthcare because of their poor health status (4). However, higher healthcare expenses may make them choose not to see a doctor when they are sick (5). To meet the requirements of low-income people for basic healthcare and reduce their medical financial burden in rural areas, China began piloting medical financial assistance (MFA) in 2003. MFA achieved full coverage in rural areas in 2008 and to assure some coverage for patients with serious diseases in 2012. As the last line of defense for health security, the impact of MFA on low-income people's healthcare expenses and medical financial burden in rural areas of China needs to be further explored.

The Interim Measures for Social Assistance implemented in 2014 pointed out that MFA mainly covers “Dibao,” “Wubao,” and “Tekun” households, which are also regarded as low-income households in rural China. “Dibao” households are those whose per capita monthly household income is below a specific standard. The “Wubao” and “Tekun” households include the elderly, disabled, and minors who are unable to work, have no source of income, and have no dependents. The subsidy system of MFA is designed as follows: on the one hand, MFA subsidizes the insurance premium for low-income people so that they can be covered by health insurance before they get sick; on the other hand, health insurance does not reimburse all healthcare expenses, and the insured must copay for healthcare costs because of moral hazard. Against this backdrop, MFA also subsidizes a percentage of out-of-pocket healthcare expenses (OOPE) for low-income people after medical insurance reimbursement (6). Therefore, MFA will release the healthcare demand and provide financial protection to low-income people in rural China if it operates well.

In recent years, studies on the impact of MFA on healthcare accessibility and the medical financial burden based on data from MFA pilot regions have gradually emerged in China. Previous studies consistently found that MFA significantly improves access to healthcare for low-income people (5, 7–10). However, evidence on the impact of MFA on the medical financial burden is mixed. For example, Dai et al. (1) found that MFA significantly reduces the medical financial burden on people with low income, while Liu et al. (11) found no significant effect of MFA on the medical financial burden.

The variability in the previous literature on the impact of MFA on the medical financial burden can be attributed to two aspects. On the one hand, MFA and health insurance play an insufficient role in reducing the medical financial burden. The functionality of MFA is partially dependent on health insurance. Health insurance can improve access to healthcare by sharing a certain percentage of medical expenses of the insured (7–10, 12). However, health insurance also encourages the insured to seek care from higher-level providers and pushes up their OOPE (13–15). Combined with limited subsidies for OOPE from MFA, low-income people still face a heavy medical financial burden. On the other hand, the results obtained using different data and methods are also significantly different. In previous studies, the data sources used were quite different. For example, the data in Fang's study came from three counties in the eastern, central, and western regions of China (16). The data in Dai's study came from Lishui, a city in Zhejiang province (1). This difference also exists in the methods used in the rest of the existing literature.

The existing literature has provided important references for this study. However, the references still have the following limitations. First, the existing literature on this subject presents conflicting results and limits the utilized samples to a certain district rather than nationwide. Considering that each district has its own characteristics, the results from these districts may not represent the overall situation in China. Second, a large number of studies have mostly used descriptive statistics for analysis. Few of them have used a more rigorous method to quantify the relationship between MFA and the medical financial burden. In addition, previous studies have failed to address the self-selection bias. It is well known that MFA beneficiaries are not randomly selected. According to the design of the MFA system in China, the issue of whether to receive medical cash assistance depends on an individual's working ability and household income; it is also considered a self-selection behavior.

This study aimed to explore the impact of MFA on healthcare expenses and the medical financial burden in rural areas of China. To overcome the limitations of previous studies, we investigated the impact of MFA by using nationally representative microdata. In addition, we also applied the propensity score matching (PSM) method to reduce potential model specification and self-selection bias. This study contributes to the growing literature on the impact of MFA on the medical financial burden by providing nationwide empirical evidence from rural China.

## Methods

### Propensity score matching

In this study, we employed PSM to estimate the impact of MFA on healthcare expenses and the medical financial burden. As a counterfactual estimation method, PSM was put forward by Rosenbaum and Rubin (17) and can be used to estimate the net effect on outcomes between treatment and control groups. The reason we used PSM to estimate the impact of MFA is to reduce potential model specification and self-selection bias. On the one hand, unlike ordinary multiple regression methods, PSM does not depend on a specific functional form, which reduces the potential model specification bias. On the other hand, whether to receive MFA is a self-selection behavior that depends on an individual's working ability, household income, and demographic characteristics. In this study, the treatment group is comprised of those with MFA coverage, while the control group is comprised of those without MFA coverage. PSM allows us to obtain the impact of MFA on the medical financial burden by matching treatment and control groups based on a series of observable factors. In other words, PSM allows us to construct a counterfactual state in which the control group is similar to the situation when the treatment group is not covered by MFA. Under this situation, the differences in the medical financial burden between the treatment and control groups are the outcomes of interest.

To perform the PSM, the observations' propensity scores being assigned to the treatment group are first estimated. Several matching methods are available to match treatment and control groups based on propensity scores, such as one-to-one matching, *k*-nearest neighbor matching, radius matching, and kernel matching methods. To date, there have been no definite criteria for determining which matching method is more accurate and efficient. The existing studies usually use different matching methods to verify the robustness of the estimated results. To test the matching quality, we employed a balance test to estimate the bias before and after matching. If the matching quality is good, the background characteristics between the treatment and control groups would be as similar as possible after matching, and the average treatment effect on the treated (ATT) would be obtained. The ATT can be estimated as follows:


(1) $$\begin{array}{l} \text{AT}{\text{T}}_{\text{PSM}}=E_{p\left(x_{i}\right)|D_{i}=1}\{E\left[Y_{i}\left(1\right)|D_{i}=1,P\left(X_{i}\right)\right]\\ \;-E\left[Y_{i}\left(0\right)|D_{i}=0,P\left(X_{i}\right)\right]\}, \end{array}$$

where *D* is a dummy variable indicating whether the respondent is covered by MAF (1 is the treatment group, 0 is the control group), *Y*_*i*_(0) is the medical financial burden on the control group, *Y*_*i*_(1) is the medical financial burden on the treatment group, and *X*_*i*_ is a covariate.

### Data

The data used in this study were derived from the 2014 China Family Panel Studies (CFPS). The CFPS is a nationally representative longitudinal tracking survey conducted by the Institute of Social Science Survey (ISSS) of Peking University. The CFPS subsample was obtained by a three-stage (districts/counties–villages/communities–households) probability of random sampling, and the samples cover 621 villages/communities from 25 of China's 30 provinces. The dataset includes rich information on respondents' health, healthcare expenses, demographic, household, and community characteristics. CFPS has been conducted nationwide since 2010 and is repeated every 2 years. Considering that only 2014 data are available to identify “Dibao,” “Wubao,” and “Tekun” households in rural areas, we use the CFPS 2014 data to analyze the impact of MFA on healthcare expenses and the medical financial burden. Respondents with non-agricultural household registrations were excluded because we only focused on low-income people in rural areas. In addition, respondents below the age of 16 were also excluded because minors do not make independent decisions regarding healthcare utilization. During this process, the sample size changes as follows: the 2018 adult data included 29,487 respondents; after deleting respondents with non-agricultural household registration, 21,343 respondents were left. After excluding respondents with missing information on selected variables, the remaining sample size was 9,022.

### Dependent variables

The dependent variables in this study were healthcare expenses and medical financial burden. According to Zhou's study (8), healthcare expenses were measured by the total healthcare expenditure in the past year (including outpatient, inpatient, drug purchase, and rehabilitation expenditures). According to the World Health Organization (WHO) definition of the medical financial burden, we measured the medical financial burden as the ratio of the respondents' OOPE to their per capita household non-food expenditure (2). In addition, we also used catastrophic healthcare expenditures (CHE) as a proxy variable for the medical financial burden. A large body of literature used 40% as the threshold at which CHE occurs, i.e., when the ratio of OOPE to per capita household non-food expenditure is greater than threshold. In this study, we also used 40% as the CHE threshold and replace it with 20% to perform robustness tests.

### Independent variables

The main independent variable was whether the respondent is covered by MFA. In rural China, MFA provides cash assistance to members of “Dibao,” “Wubao,” and “Tekun” households when they incur large healthcare expenditures. The CFPS questionnaire asks respondents to answer questions about whether their households receive government subsidies, or not, and the answers include the following eight options: “Dibao,” “subsidy for returning farmland to forest,” “agricultural subsidies,” “Wubao,” “Tekun,” and “work injury pension.” We coded respondents who receive “Dibao,” “Wubao,” and “Tekun” subsidies as 1, and we coded respondents who receive other subsidies or do not receive any subsidies as 0.

### Control variables

Healthcare expenses and the medical financial burden were also influenced by a series of individual demographic factors, household characteristics, and socioeconomic factors (18, 19). In the model, we controlled for age, gender, marriage, total household income, education, region, chronic diseases, and self-rated health. The definitions of each variable were as follows: age was measured by subtracting the year of birth from the survey year and was categorized into three stages: 0 = 16–35 years old, 1 = 36–59 years old, and 2 = 60 years old and above. In terms of gender, men were coded as 1 and women were coded as 0. Marriage was dichotomously recoded as with a spouse (1 = married or cohabitated) or single (0 = single or divorced or widowed). Household income was measured by quintiles of the total net household income (on a scale ranging from 0 to 4). Family size was assessed by the number of family members. Education is categorized as low (0 = primary school and below), middle (1 = junior and senior high school), and high (2 = college and above). Health insurance was a dummy variable indicating whether respondents are covered by health insurance (1 = yes, 0 = no). Medical institution was measured by the square of the largest medical institution in the village or community. Region was divided into three parts: east (=0), center (=1), and west (=2). Chronic disease was a dummy variable representing whether respondents have suffered from a chronic disease in the past half year (1 = yes, 0 = no). Self-rated health was assessed on a scale of 1 to 5, and the answers were dichotomously recoded into good (1 = excellent or very good or good or fair) or bad (0 = bad).

## Results

### Descriptive statistics

Table 1 presents the descriptive statistics of the variables between beneficiaries and non-beneficiaries of MFA. With respect to the sample size, 1,131 respondents are beneficiaries of MFA and 7,891 respondents are non-beneficiaries. The difference in sample size between the treatment and control groups was sufficient for the application of the PSM method. In terms of healthcare expenses and the medical financial burden, there were significant differences between the two groups. Specifically, compared with non-beneficiaries, beneficiaries tended to have a higher healthcare expense and a heavier medical financial burden. Significant differences in dependent variables indicated that MFA is associated with an increase in healthcare expenses and the medical financial burden. This finding was potentially attributed to the fact that beneficiaries have a higher demand for healthcare services and their income cannot cover their healthcare expenditures. Significant differences also existed in most of the control variables between beneficiaries and non-beneficiaries. It should be noted that beneficiaries have a worse status in regard to health, such as self-rated health and the prevalence of chronic diseases, compared to non-beneficiaries. At the same time, more than half of the beneficiaries' household income were below the middle level, which is the opposite to that of non-beneficiaries. In addition, beneficiaries were older in age and younger in other socioeconomic characteristics, such as marriage, education, health insurance, and medical institutions. More than half of the beneficiaries live in the central and western regions of China. However, differences in healthcare expenses and the medical financial burden cannot be directly attributed to MFA because such results require empirical testing. Therefore, we used the PSM method to further explore the relationship between MFA and healthcare expenses and the medical financial burden.

**Table 1:** The descriptive statistics of variables.

**Variables, *n* (%) or mean (SD)**	**Beneficiaries**	**Non-beneficiaries**	**Diff/Chi^2^**	***P Value***
Healthcare expense	3,925.038 (12,911.620)	2,741.242 (8,987.296)	−1,183.796	0.001
Ratio of OOPE to expenditure	0.582 (1.777)	0.419 (2.502)	−0.163	0.034
Catastrophic healthcare expenditures (40%)			26.180	0.000
Yes	345 (30.50%)	1,866 (23.65%)		
No	786 (69.50%)	6,025 (76.35%)		
Catastrophic healthcare expenditures (20%)				
Yes	523 (46.24%)	3,000 (38.02%)		
No	608 (53.76%)	4,891 (61.98%)		
Age			60.600	0.000
16–35	247 (21.84%)	2,077 (26.32%)		
36–59	495 (43.77%)	3,913 (49.59%)		
60+	389 (34.39%)	1,901 (24.09%)		
Gender			13.279	0.000
Men	593 (52.43%)	3,666 (46.46%)		
Women	538 (47.57%)	4,225 (53.54%)		
Marital status			81.644	0.000
Married/cohabiting	823 (72.77%)	6,576 (83.34%)		
Single/divorced/widowed	308 (27.23%)	1,315 (16.66%)		
Family size	4.469 (2.000)	4.498 (1.864)	0.028	0.639
Household income			217.837	0.000
Lowest	466 (41.20%)	1,933 (24.50%)		
Second	293 (25.91%)	1,661 (21.05%)		
Third	162 (14.32%)	1,561 (19.78%)		
Fourth	114 (10.08%)	1,547 (19.60%)		
Highest	96 (8.49%)	1,189 (15.07%)		
Education			80.125	0.000
Primary school and below	822 (72.68%)	4,691 (59.45%)		
Junior/senior high school	298 (26.35%)	3,047 (38.61%)		
College and above	11 (0.97%)	153 (1.94%)		
Medical institution	254.693 (643.631)	248.334 (709.316)	−6.359	0.775
Region			132.645	0.000
East	255 (22.55%)	3,014 (38.20%)		
Central	352 (31.12%)	2,305 (29.21%)		
West	524 (46.33%)	2,572 (32.59%)		
Self-rated health			91.979	0.000
Good	751 (66.40%)	6,244 (79.13%)		
Bad	380 (33.60%)	1,647 (20.87%)		
Chronic disease			5.651	0.017
Yes	283 (25.02%)	1,721 (21.81%)		
No	848 (74.98%)	6,170 (78.19%)		
Sample size	1,131	7,891		

Continuous variables are analyzed by two-independent-sample t-test. Dummy variables are analyzed by a chi-squared test.

### The matching quality of PSM

The premise of using the PSM method to obtain ATT depends on the matching quality. If there were no significant differences in background characteristics between beneficiaries and non-beneficiaries after matching, then the matching quality is good. In this study, matching quality was assessed by changes in a series of parameters before and after matching, such as mean bias and the degree of bias reduction between the treatment and control after matching. Table 2 shows that the mean bias between the treatment and control groups on a series of covariates is reduced to < 10 after matching. The *t*-value also shows that differences in covariates between the treatment and control groups change from significant before matching to insignificant after matching. Changes in a series of parameters before and after matching indicate that the matching quality of PSM in this study is good.

**Table 2:** The matching quality of the propensity score matching (PSM) model.

**Variables**	**Matching status**	**Treated (mean)**	**Control (mean)**	**Bias (%)**	**Reduction bias (%)**	***T Value***
Age	Unmatched	1.126	0.978	20.4		6.52
	Matched	1.126	1.122	0.5	97.6	0.11
Gender	Unmatched	0.524	0.465	12.0		3.77
	Matched	0.524	0.486	7.6	36.4	1.81
Marital status	Unmatched	0.728	0.833	-25.7		-8.69
	Matched	0.728	0.747	-4.7	81.6	-1.05
Family size	Unmatched	4.470	4.498	-1.5		-0.47
	Matched	4.470	4.520	-2.6	−79.8	-0.61
Household income	Unmatched	1.187	1.797	-45.2		-13.85
	Matched	1.187	1.170	1.3	97.1	0.33
Education	Unmatched	0.283	0.425	-28.2		-8.51
	Matched	0.283	0.282	0.2	99.4	0.05
Medical institution	Unmatched	254.690	248.330	0.9		0.29
	Matched	254.690	250.430	0.6	33.0	0.14
Region	Unmatched	1.238	0.944	35.9		11.08
	Matched	1.238	1.261	-2.8	92.2	-0.68
Self-rated health	Unmatched	0.664	0.791	-28.9		-9.64
	Matched	0.664	0.652	2.8	90.3	0.62
Chronic disease	Unmatched	0.250	0.218	7.6		2.43
	Matched	0.250	0.239	2.7	64.2	0.64

### The impact of MFA on healthcare expenses

Table 3 presents the ATT of healthcare expenses after matching. From this table, it is clear that the average healthcare expense of MFA beneficiaries is higher than that of non-beneficiaries. This result is robust across all four different matching methods. The ATT values under the different matching methods are more than RMB ¥770 ($110). The increase in healthcare expenses also indicates a rise in healthcare utilization. Therefore, MFA releases the healthcare demand of low-income people and promotes their healthcare utilization by enrolling them in health insurance and providing them with medical cash assistance in rural China. Moreover, MFA also reduces income-related inequality in healthcare utilization, which may benefit the health of low-income people with chronic or serious diseases (20). However, it is worth noting that the release of healthcare demands is not the goal of MFA. Although low-income people enjoy reasonable healthcare services, their medical financial burden also needs to be further reduced.

**Table 3:** The impact of MFA on healthcare expenses.

**Matching method**	**Common support sample size**	**Total healthcare expense**			**SE**	***T value***
		**Treatment**	**Control**	**ATT**		
One-to-one matching (*k* = 1)	8,999	3,925.038	3,014.331	910.707^*^	555.361	1.95
K-nearest neighbor matching (*k* = 10)	8,999	3,925.038	3,047.655	877.383^**^	466.382	2.12
Radius matching (δ = 0.01)	8,987	3,928.246	3,157.185	771.061^*^	400.252	1.89
Kernel matching (k: epan bw: 0.06)	8,999	3,925.038	3,118.025	807.013^**^	395.724	2.00

ATT, average treatment effect on the treated; K, number of neighborhoods for matching; δ, matching radius; epan, kernel function; bw, bandwidth.

* *p* < 0.1;

** *p* < 0.05; ^***^p < 0.01.

### The impact of MFA on medical financial burden

Table 4 presents the results of the impact of MFA on the medical financial burden after matching. From the results, it is clear that the ATT of one-to-one matching, *k*-nearest neighbor matching, and kernel matching methods are all positive. In addition, the *t*-values show that the ATT of all matching methods is not significant. The results in Table 4 indicate that MFA has no significant impact on the medical financial burden on low-income people in rural areas of China. The ratio of OOPE to per capita household non-food expenditure of MFA beneficiaries is still higher than that of non-beneficiaries after matching, indicating that the medical financial burden on low-income people remains heavy.

**Table 4:** The impact of MFA on the medical financial burden.

**Matching method**	**Common support sample size**	**Ratio of OOPE to expenditure**			**SE**	***T Value***
		**Treatment**	**Control**	**ATT**		
One-to-one matching (*k* = 1)	8,999	0.582	0.472	0.110	0.168	1.59
K-nearest neighbor matching (*k* = 10)	8,999	0.582	0.574	0.008	0.127	0.11
Radius matching (δ = 0.01)	8,987	0.582	0.591	−0.008	0.090	−0.13
Kernel matching (k: epan bw: 0.06)	8,999	0.582	0.573	0.009	0.086	0.14

ATT, average treatment effect on the treated; K, number of neighborhoods for matching; δ, matching radius; epan, kernel function; bw, bandwidth.

### Robustness tests on the medical financial burden

In this section, we replaced the ratio of OOPE to per capita household non-food expenditure with CHE to perform robustness tests on the impact of MFA on the medical financial burden. As presented in Table 5, when the CHE threshold was set at 40%, all ATT values were positive but not significant. The result was similar when the threshold of CHE was set at 20%. Although the ATT values were not significant, MFA beneficiaries are more likely to incur CHE than non-beneficiaries. Therefore, MFA does not reduce the medical financial burden on low-income people in rural China. This result was robust across different matching methods and different medical financial burden indicators. This result was also contrary to the theoretical analysis and the original intention of MFA.

**Table 5:** The impact of MFA on catastrophic healthcare expenditures (CHEs).

**Matching method**	**Probability of CHE (40%)**				**Probability of CHE (20%)**			
	**ATT**	**SE**	***T value***	**Sample size**	**ATT**	**SE**	***T value***	**Sample size**
One-to-one matching (*k* = 1)	0.015	0.025	0.71	8,999	0.012	0.025	0.53	8,999
K-nearest neighbor matching (*k* = 10)	0.015	0.016	0.97	8,999	0.017	0.020	0.99	8,999
Radius matching (δ = 0.01)	0.012	0.015	0.80	8,987	0.016	0.017	0.95	8,987
Kernel matching (k: epan bw: 0.06)	0.017	0.013	1.12	8,999	0.023	0.016	1.41	8,999

ATT, average treatment effect on the treated; K, number of neighborhoods for matching; δ, matching radius; epan, kernel function; bw, bandwidth.

## Discussion

This study aimed to explore the impact of MFA on low-income people's healthcare expenses and medical financial burden in rural areas of China, which is a key area of inequality in healthcare utilization in China at the moment. The results obtained in this study indicate that MFA has made some achievements in improving access to healthcare, including outpatient and inpatient services. The implementation of MFA increases the likelihood of low-income people to consume healthcare services, which indicates that MFA releases their healthcare demands. However, the impact of MFA is not significant in reducing the medial financial burden on low-income people. As a result, low-income people still face a heavy financial burden after seeking healthcare in rural China. Thus, the role of MFA should be further improved.

One of the key findings of this study was that MFA increases the likelihood that low-income people will consume healthcare services. This finding was consistent with previous studies on the effect of health insurance, which have suggested that health security policy has a positive effect on healthcare utilization (8–10, 21–23). Possible reasons for the positive impact on healthcare utilization by low-income people are mainly economic incentives. For example, in the past, medical financial barriers were a key limitation on access to healthcare in rural China; low-income people did not dare to seek healthcare when they were sick. After the implementation of MFA, the price of healthcare faced by low-income people will decrease, as MFA not only subsidizes insurance costs so that they will be covered by the NCMS, but also provides direct cash assistance for their OOPE. According to the price elasticity of demand theory, a decrease in the price of healthcare will release people's healthcare demand and encourage them to consume more healthcare services. Generally, the price elasticity of routine healthcare services is greater than that of non-routine healthcare services, as the former has strong substitutability. Diseases associated with non-routine healthcare services are usually life-threatening, and people have no further options. However, the price elasticity of non-routine healthcare services for low-income people is also great because they will not choose treatment when they cannot afford healthcare expenses. Therefore, in rural China, low-income people choose to go to a doctor when the NCMS partially reimburses their healthcare expenses and MFA partially subsidizes their OOPE.

Another finding of this study shows that MFA does not significantly reduce the medical financial burden on low-income people, which is contrary to the principles of MFA. This result is similar to the findings of previous studies. The results from existing studies also confirm that increases in health insurance and MFA coverage have not been accompanied by reductions in the medical financial burden (4, 7, 13, 16, 24). There are two possible explanations for the negligible impact of MFA on reducing the medical financial burden. On the supply side, healthcare quality provided by primary care facilities is usually low due to talent and medical technology-related issues. The unbalanced allocation of healthcare resources between primary care facilities and large hospitals makes patients to bypass primary care and access healthcare at higher-level hospitals when they need medical treatment (25). Therefore, the imperfection of the hierarchical medical system also increases patients' healthcare expenditure. On the demand side, the availability of MFA also enables low-income people to take on healthcare expenses that they know will be very high and potentially catastrophic, but which they are willing to take on because they need the care. This may be treatment for a past illness, improved management of existing chronic diseases, or non-urgent but important care they need now. In addition, they also know that they will receive some financial support in this process as their healthcare expenditures will be subsidized by health insurance and medical cash assistance. However, health insurance and MFA cannot cover all of their healthcare expenditures, and the overall subsidy level of MFA remains low. Moreover, indirect healthcare expenditures that are part of OOPE are also not covered by health insurance and MFA, such as travel and accommodation expenses.

This study contributes to the literature on the impact of MFA by providing more quantitative evidence from China. In this study, we initially quantified the relationship between MFA and healthcare expenses and the medical financial burden using a national sample from China. Specifically, we employed the PSM method to reduce potential model specification bias and sample self-selection problems and provide more accurate assessments of the financial incentive for MFA. It is interesting to policymakers that MFA does not significantly reduce the medical financial burden on beneficiaries. It is well-known that China has entered an “era of post-poverty alleviation.” How to prevent people from falling back into poverty due to diseases has become a more serious policy issue in China. In addition, guaranteeing people's access to basic healthcare services without financial barriers is also a requirement of the Healthy China strategy. Against this backdrop, the government should focus on how to raise the security level of MFA to reduce the financial burden on low-income people after they seek healthcare.

Some people may doubt the timeliness of the data used in this study. The reason we used 2014 data is that the CFPS only collected respondents' MFA information in the 2014 survey. Although the CFPS completed its fifth national tracking survey in 2018, we were unable to accurately identify “Dibao,” “Wubao,” and “Tekun” households in the 2018 data. Specifically, the CFPS 2018 questionnaire asked respondents whether they received government subsidies in the past 12 months, the answers included “Dibao,” “agricultural subsidy,” “subsidy for returning farmland to forest,” “Wubao,” and “Tekun.” CFPS coded the respondents who received any of these subsidies as “1” and the rest as “0.” As a result, it is difficult to identify the respondents who only received subsidies for “Dibao,” “Wubao,” and “Tekun.”

The security levels of MFA and health insurance may change between 2014 and 2018. For example, the MFA reimbursement rate for OOPE was 70% in 2014 but was adjusted to 80% in Beijing in 2018. In terms of health insurance, the per capita financial subsidy standard for health insurance also increased from RMB ¥320 ($45.92) in 2014 to RMB ¥490 ($70.3) in 2018. Against this backdrop, we matched the CFPS 2014 data to the 2018 data and identified respondents who participated in both surveys. We assumed that MFA beneficiaries were the same between 2014 and 2018 and explored the impact of MFA on the medical financial burden using the matched 2018 data. The result also shows that MFA does not significantly reduce the medical financial burden on low-income people. However, caution should be applied to the result derived from the 2018 data in that MFA beneficiaries in 2014 may not have received MFA coverage in 2018. Overall, although the data used are from 2014, this study initially quantifies the impact of MFA using nationally representative data. The results of this study also form a basis for decision-making to improve MFA to a certain extent.

If the latest national survey data set was established and includes respondents' MFA information, further research should be undertaken to evaluate the impact of MFA using the latest data. This would provide more important evidence of the continuous improvement of MFA.

## Conclusion

The findings of this study showed that MFA has released the healthcare demand of low-income people and encouraged them to seek healthcare in case of illness. Specifically, MFA beneficiaries' total healthcare expenditure has increased significantly to some extent. However, MFA plays a limited role in terms of the medical financial burden. Although beneficiaries' healthcare expenditures can be proportionally subsidized by health insurance and medical cash assistance, they still face a heavy medical financial burden. Therefore, policymakers need to take measures to reduce the medical financial burden on low-income people. The government should gradually raise MFA standards for rural areas to achieve equal treatment between urban and rural areas. In addition, it is also important to improve the MFA system for serious diseases and expand its coverage to include the accumulated chronic healthcare expenditure during the year. Moreover, the coordination between MFA and health insurance needs to be strengthened such that reasonable healthcare expenditures incurred outside the health insurance catalog are subsidized by MFA.

## Reflexivity statement

The coordination between MFA and health insurance needs to be strengthened, such as reasonable healthcare expenditure incurred outside the health insurance catalog should also be subsidized by MFA.

## Data availability statement

The datasets presented in this study can be found in online repositories. The names of the repository/repositories and accession number(s) can be found below: http://www.isss.pku.edu.cn/cfps/. Stata Code is available from the corresponding author upon reasonable request.

## Author contributions

YC: conceptualization, design, analyzing data, and writing original draft. GG and FY: review and re-edit. YZ: writing original draft and review. All authors contributed to the article and approved the submitted version.
